# HAI and NAI titer correlates of inactivated and live attenuated influenza vaccine efficacy

**DOI:** 10.1186/s12879-019-4049-5

**Published:** 2019-05-22

**Authors:** Peter B Gilbert, Youyi Fong, Michal Juraska, Lindsay N Carpp, Arnold S Monto, Emily T Martin, Joshua G Petrie

**Affiliations:** 1 Department of Biostatistics, Bioinformatics, and Epidemiology, Vaccine and Infectious Disease Division, Fred Hutchinson Cancer Research Center, 1100 Fairview Ave. N., Seattle, 98109 USA; 2 Department of Biostatistics, University of Washington, 1705 NE Pacific St., Seattle, 98195 USA; 3 Department of Epidemiology, University of Michigan School of Public Health, 1415 Washington Heights, Ann Arbor, 48109 USA

**Keywords:** FLUVACS trial, Hemagglutinin inhibition (HAI) titers, Immune correlates, Neuraminidase inhibition (NAI) titer, Principal stratification/vaccine efficacy moderation framework, Vaccine efficacy

## Abstract

**Background:**

High hemagglutination inhibition (HAI) and neuraminidase inhibition (NAI) titers are generally associated with reduced influenza risk. While repeated influenza vaccination reduces seroresponse, vaccine effectiveness is not always reduced.

**Methods:**

During the 2007-2008 influenza season, a randomized, placebo-controlled trial (FLUVACS) evaluated the efficacies of live-attenuated (LAIV) and inactivated influenza vaccines (IIV) among healthy adults aged 18-49 in Michigan; IIV vaccine efficacy (VE) and LAIV VE against influenza disease were estimated at 68% and 36%. Using the principal stratification/VE moderation framework, we analyzed data from this trial to assess how each VE varied by HAI or NAI responses to vaccination observed for vaccinated individuals and predicted counterfactually for placebo recipients. We also assessed how each VE varied with pre-vaccination/baseline variables including HAI titer, NAI titer, and vaccination history.

**Results:**

IIV VE appeared to increase with Day 30 post-vaccination HAI titer, albeit not significantly (*p*=0.20 and estimated VE 14.4%, 70.5%, and 85.5% at titer below the assay lower quantification limit, 512, and 4096 (maximum)). Moreover, IIV VE increased significantly with Day 30 post-vaccination NAI titer (*p*=0.040), with estimated VE zero at titer 10 and 92.2% at highest titer 640. There was no evidence that fold-change in post-vaccination HAI or NAI titer associated with IIV VE (*p*=0.76, 0.38). For LAIV, there was no evidence that VE associated with post-vaccination or fold-rise HAI or NAI titers (p-values >0.40). For IIV, VE increased with increasing baseline NAI titer in those previously vaccinated, but VE decreased with increasing baseline NAI titer in those previously unvaccinated. In contrast, for LAIV, VE did not depend on previous vaccination or baseline HAI or NAI titer.

Conclusions: Future efficacy trials should measure baseline and post-vaccination antibody titers in both vaccine and control/placebo recipients, enabling analyses to better elucidate correlates of vaccine- and natural-protection.

Trial registration: ClinicalTrials.gov NCT00538512. October 1, 2007.

**Electronic supplementary material:**

The online version of this article (10.1186/s12879-019-4049-5) contains supplementary material, which is available to authorized users.

## Background

Influenza hemagglutinin (HA) binds to sialic acid receptors on target cells and is the main target of neutralizing antibodies [1]. As such, HA is the primary standardized antigen in the inactivated influenza vaccine (IIV) [2]. Serum levels of anti-HA antibodies are measured by the hemagglutination inhibition (HAI) assay. When assay targets are well matched to circulating viruses, there is generally a clear relationship between increasing HAI titer and decreasing infection risk [3–9]; however, some individuals acquire infection or experience influenza disease despite high HAI titers [4, 10–12] or seroconversion [12]. Neuraminidase (NA) is also present in most influenza vaccines, albeit at nonstandardized concentrations [13]. As measured by neuraminidase inhibition (NAI) assays, anti-NA antibodies play an independent role in protection from influenza disease and/or in reducing influenza disease severity [14, 15].

Antigenic drift [16–18] necessitates annual evaluation of influenza vaccine composition [19]. To optimize the immune response to strains most likely to cause infection, annual influenza vaccination is currently recommended in the United States for all individuals aged ≥6 months (unless contraindicated) [20]. However, reduced seroresponse can occur after repeated influenza vaccination [21–26]. While this suggests that repeated vaccination may have diminishing benefit to protect against influenza infection or disease, reduced vaccine effectiveness with repeated vaccination has only been observed in some, primarily A(H3N2) predominant, seasons [27–35]. These apparently inconsistent results could be explained by the degree of antigenic relatedness between vaccine and circulating viral strains [36, 37].

The principal stratification/vaccine efficacy (VE) moderation framework [38–40] (hereafter called the “VE moderation” framework) is a statistical method for assessing how vaccine efficacy varies over subgroups defined by biomarkers measuring immune responses in vaccinees. This framework requires data from a randomized-controlled trial with sufficient endpoint cases and ample immune response measurements from vaccinees in cases and non-cases, as well as variables measured at baseline in both vaccine and placebo recipients (cases and non-cases) that are predictive of post-vaccination immune responses. The latter requirement enables the baseline immunogenicity predictor (BIP)-augmented efficacy trial design [39, 40] that predicts the immune response to the vaccine that placebo recipients would have had had they been vaccinated, allowing estimation of the VE-by-postvaccination-titer curve. Using this framework, fold-rise in anti-varicella zoster virus (VZV) titers was shown to be strongly associated with VE against herpes zoster disease, whereas post-vaccination titers at 6 weeks were not, implying that baseline titers must be measured to predict VE [41]. This framework also showed that post-vaccination neutralization titers were strongly associated with VE against virologically confirmed dengue [42]. We applied this framework to data collected as part of a randomized, placebo-controlled trial of the absolute and relative efficacies of IIV and live-attenuated influenza vaccine (LAIV) [43] to assess how VE against laboratory–confirmed influenza disease varied across subgroups defined by their HAI or NAI responses to vaccination. We also studied how VE varied with baseline/pre-vaccination data on HAI, NAI, and relevant clinical variables.

## Methods

### Study design and intervention

The FLUVACS trial enrolled healthy adults aged 18 to 49 years, excluding individuals with a health condition for which vaccination was specifically recommended (including being immunocompromised or being older than 49 years) or for which either vaccine was contraindicated. Participants were recruited from October to November 2007 and randomly assigned to receive IIV (Fluzone, Sanofi Pasteur), LAIV (FluMist, MedImmune), or saline placebo. Surveillance for influenza-like illness was carried out from November 2007 through April 2008. Overall IIV and LAIV efficacy were 68% and 36%, respectively [43].

### Influenza endpoint, cases and controls

The study endpoint was laboratory–confirmed influenza disease, defined as symptomatic acute respiratory illness subsequently confirmed by RT-PCR influenza virus identification [43]. Participants with an observed endpoint are referred to as cases and participants completing follow-up (with a post-season sample) without experiencing the endpoint are referred to as controls.

For antibody response measurements, serum samples were collected at Day 0 (immediately before intervention administration), Day 30 (approximately), and at the influenza season conclusion (approximately 4 months later). HAI titers were measured in a subset of all participants (including participants in each treatment arm) consisting of all cases and a random sample of participants for whom all 3 serum samples were available [14]. NAI titers were measured in all cases and in a smaller sub-sample of controls [14].

### HAI and NAI titer variables

The HAI assay measures the highest dilution of serum that prevents influenza virus-induced hemagglutination of erythrocytes [44]. The reciprocal of this dilution was defined as the HAI titer. In the lectin-based NAI assay [45], the reciprocal of the highest dilution of serum that inhibits NA activity at least 50% compared to control wells was defined as the NAI titer. Titers below the lower limit of quantification for both assays were set to half this value [14]. For HAI titers in graphs, *log*_2_(*x*) for *x*=0,1,2, etc. corresponds to titer (2^*x*^)∗4, and for NAI titers *log*_2_(*x*) corresponds to titer (2^*x*^)∗5. Fold-rise in HAI or NAI titer was defined as (Day 30 titer)/(Day 0 titer).

### Vaccine efficacy parameters

Overall vaccine efficacy (VE) for either vaccine versus placebo was defined as the multiplicative reduction in the probability of influenza disease occurrence:

$$\text{VE} = 1 - \frac{P(\text{influenza disease}|\text{vaccine})}{P(\text{influenza disease}|\text{placebo})}.$$

VE for a vaccinated subgroup defined by a fixed value *s* of the Day 30 or fold-rise in titer was defined as

$$\text{VE}(s) = 1 - \frac{P(\text{influenza disease}|\text{vaccine, titer} s)}{P(\text{influenza disease}|\text{placebo, titer} s)}.$$

The critical feature of the VE moderation framework is that the subgroup *s* is defined under assignment to a vaccine group, which is observable for participants actually assigned to a vaccine group, but is counterfactual and hence missing for participants assigned to the placebo group [39, 40, 46]. Implementation of this framework for estimating VE over subgroups *s* requires prediction of the missing counterfactual responses of placebo recipients [41].

For studying the association of baseline variables with VE, the VE parameter of interest is one minus the ratio (vaccine/placebo) of the disease incidence in subgroups defined by fixed values of the baseline variables.

### Objectives

We investigated: (1) if VE varies by Day 0 HAI or NAI titer and/or by other baseline clinical variables; (2) if VE varies by fold-rise HAI titer and by fold-rise NAI titer; (3) if VE varies by Day 30 HAI titer and by Day 30 NAI titer. Baseline clinical variables included age, sex, race, and whether the individual self-reported ever having received an influenza vaccine before (hereafter referred to as “previously vaccinated”).

### Statistical analysis

Boxplots and scatterplots with Spearman rank correlations describe the Day 0, 30, and fold-rise HAI and NAI titer distributions. These distributions were compared across groups (treatment arms, vaccination history) using Wilcoxon rank-sum tests, and across age levels by Spearman rank correlations.

Objective (1) was addressed by multivariable logistic regression (with sandwich variance estimates) modeling of how the risk of influenza outcome depended on titer variables and clinical variables, which is valid under the case-control sampling design [47]. Forward selection stepwise regression based on Wald tests was used to select best-fitting models.

Objectives (2) and (3) were addressed using the same or similar statistical methods as in [41]. The influenza disease endpoint was treated as a dichotomous outcome (case vs. control); time-to-event methods would not add value given all vaccinations were completed from 10/10/2007 to 11/09/2007, all influenza events occurred between 1/10/2008 and 3/09/2008, and 96.4% of participants completed all scheduled visits in this year.

For Day 30 or fold-rise variables treated as quantitative variables, the Juraska et al. method [48] was applied, which was also used in [42]. We describe the method for Day 30 HAI titer; the same method is used swapping in Day 30 NAI titer, fold-rise HAI titer, and fold-rise NAI titer. This method specifies a structural logistic regression model

$${} P(Y(z)\,=\,1|S(z)=s,X=x) \,=\, expit \left(\beta_{0z} \,+\, \beta_{1z} S(z) + \beta_{2z} X \right) $$

where *Y*(*z*) is the indicator of the influenza endpoint if assigned treatment *z*, *z*=0 (placebo) and *z*=1 (vaccine), *expit*(*a*)=*exp*(*a*)/(1+*exp*(*a*)),*S*(*z*) is Day 30 HAI titer if assigned treatment *z* (*z*=0,1), and *X* is the baseline covariate age in years at enrollment. Inverse probability-weighting is used in the logistic regression model is used to account for the probabilities participants have the titer data measured. The method incorporates an estimate of the conditional density of *S*(1) given *S*_*b*_ and *X*, obtained by nonparametric kernel smoothing with optimal bandwidths selected by likelihood cross-validation [49], where *S*_*b*_ is Day 0 HAI titer. A main assumption of this method is that the risk of *Y*(0)=1 is conditionally independent of *S*(1) given *S*(0) and *X*, which in our application states that after accounting for age and the Day 30 HAI titer if assigned placebo, Day 30 titer if assigned vaccine does not contain additional information about influenza risk if assigned placebo.

The method outputs point estimates of *VE*(*s*), bootstrap-based 95% pointwise and simultaneous confidence bands for VE (*s*), and a bootstrap-based 2-sided p-value for testing whether VE (*s*) varies in *s*, for each of the IIV and LAIV versus placebo.

For regression analyses of objectives (1)–(3), quantitative response variables were analyzed on the log_2_ scale. Analyses for objectives (2) and (3) were conducted using the pssmooth R package available at CRAN [50].

## Results

### HAI and NAI titer data

The numbers of study participants by treatment group and influenza disease case-control status, as well as by Day 0 and Day 30 HAI and NAI titer data and previous vaccination, are shown in Table 1. Of the IIV, LAIV, and placebo groups, 279 of 813 (34.3%), 324 of 814 (39.8%), and 125 of 325 (38.5%) individuals had HAI titer data at both time points. A subset of these also had NAI titer data at both time points (178, 227, and 92 individuals, respectively). Of the individuals with HAI and NAI titer data, 22, 52, and 29 (IIV, LAIV, and placebo) had the influenza disease endpoint. In these groups 676 of 813 (83.1%), 695 of 814 (85.4%), and 274 of 325 (84.3%) had data on previous vaccination, of whom 42.6%, 44.2%, and 49.6% had been previously vaccinated.

**Table 1:** HAI and NAI Titer Plus Previous Vaccination Data for the Year-4 FLUVACS Study ^∗^

			HAI Titers	NAI Titers
Treatment	Group	No.	No. w/ Day 0 and Day 30	No. w/ Day 0 and Day 30
Placebo	Total	325	125	92
	Cases	30	30	29
	Controls	295	95	63
LAIV	Total	814	324	227
	Cases	54	54	52
	Controls	760	270	175
IIV	Total	813	279	178
	Cases	22	22	22
	Controls	791	257	156
Placebo	Total	136	48	42
Restrict	Cases	15	15	15
EVERVAX=1 ^∗∗^	Controls	121	33	27
LAIV	Total	307	135	92
Restrict	Cases	26	26	24
EVERVAX=1	Controls	281	109	68
IIV	Total	288	99	58
Restrict	Cases	6	6	6
EVERVAX=1	Controls	282	93	52
Placebo	Total	138	54	35
Restrict	Cases	11	11	10
EVERVAX=0	Controls	127	43	25
LAIV	Total	388	144	105
Restrict	Cases	21	21	21
EVERVAX=0	Controls	367	123	84
IIV	Total	388	128	93
Restrict	Cases	13	13	13
EVERVAX=0	Controls	375	115	80

^∗^In the third column, all participants with an observed influenza endpoint (Cases) and all participants completing follow-up (with a post-season sample) without experiencing the influenza endpoint (Controls) are included in the counts, regardless of whether immunological data were measured. The remaining columns include the subset of these participants with HAI or NAI titer measurements at Day 0 and Day 30. For each assay HAI and NAI, all participants either had both Day 0 and Day 30 titers measured, or neither Day 0 and Day 30 titers measured. All participants with NAI titers measured also had HAI titers measured. ^∗∗^EVERVAX is self-report of ever having received a previous flu vaccine: 1 = yes, 0 = no

**Fig. 1:**
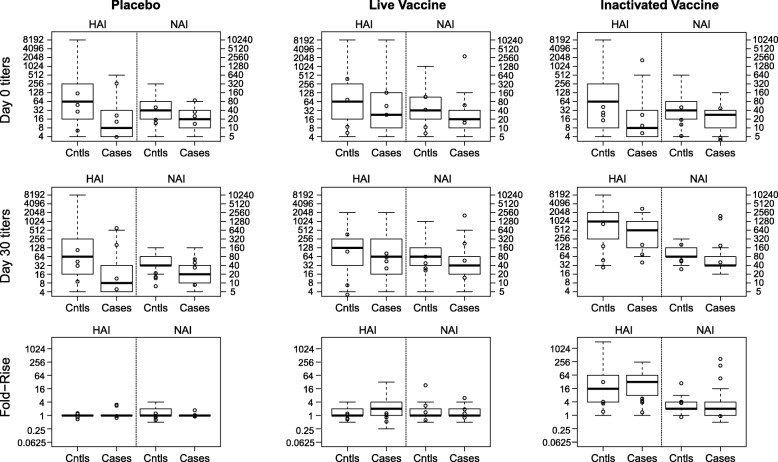
Boxplots of Day 0, Day 30, and fold-rise HAI and NAI titers by treatment group (Placebo, LAIV, IIV) and influenza case-control outcome status. Boxplots of HAI and NAI titers by treatment group and influenza case-control outcome status. Day 0 titers (top row), Day 30 titers (middle row), and titer fold-rise (Day 30 / Day 0) are shown for participants assigned to placebo (left column), live attenuated influenza vaccine (middle column), or inactivated influenza vaccine (right column)

Boxplots of Day 0, Day 30, and fold-rise HAI and NAI titers for each treatment group, stratified by case-control status, are shown in Fig. 1. Day 0 HAI and NAI titers were higher in controls than cases consistently across the three groups. This pattern also occurred for Day 30 HAI and NAI titers, with the difference between cases and controls about the same for the two vaccines and greatest for the placebo group. Fold-rises in HAI and NAI titers were similar when comparing cases and controls across the three treatment groups, with much greater variability in fold-rise responses for the vaccine groups than the placebo group. Day 30 vaccine responses were greater for the IIV than the LAIV. Moreover, fold-rise was larger for HAI compared to NAI.

Distributions and scatterplots of all pairs of Day 0, Day 30, and fold-rise HAI and NAI titer variables for each of the two vaccine groups and for the set of vaccinees with complete HAI and NAI data are shown in Additional file 1: Figures S1 and S2; corresponding plots for the placebo group are shown in Figure S3. For each assay there were moderate-to-strong direct correlations between Day 0 titers with Day 30 titers and inverse correlations between Day 0 titers with fold-rise titers, with Spearman rank correlations for the IIV 0.47, 0.68, 0.66 and 0.57 between (Day 0 HAI, Day 30 HAI), (Day 0 NAI, Day 30 NAI), (Day 0 HAI, fold-rise HAI), and (Day 0 NAI, fold-rise NAI), respectively, compared to 0.81, 0.85, 0.53, and 0.36 for the LAIV. The high correlation for Day 0 and Day 30 titers for LAIV occurs because most participants did not have antibody response to vaccination. The reasonably strong correlations between Day 0 and post-vaccination titers (Day 30, fold-rise) indicated that the baseline immunogenicity predictor-based statistical methods for estimating the VE(*s*) curves using the VE moderation framework could be effectively applied, for each vaccine and each assay. For the IIV, Day 0 titers were similarly predictive of Day 30 titers and of fold-rise titers, implying similar precision for assessing how VE varied with Day 30 titers and with fold-rise titers. In contrast, for the LAIV Day 0 titers were more predictive of Day 30 titers than fold-rise titers, implying that Day 30 titers could be assessed as a correlate of VE with more precision than fold-rise titers.

**Fig. 2:**
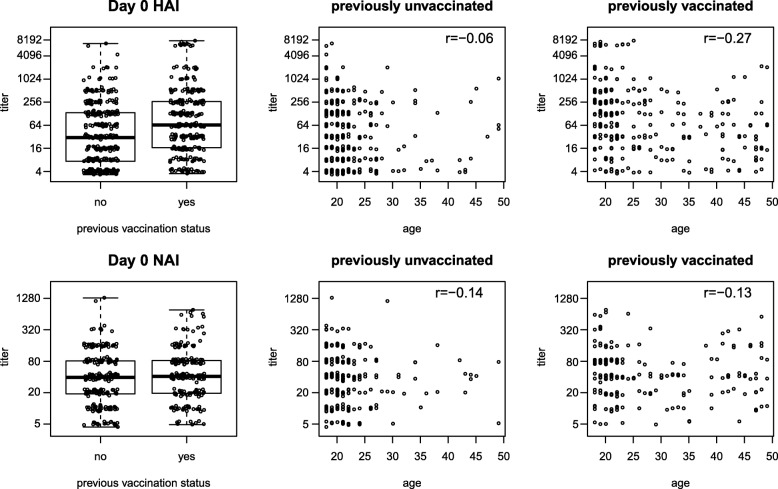
Distributions of Day 0 HAI and NAI titers by previous vaccination status and age. for all three treatment groups combined. Small perturbations are added to titer values to improve visibility. Spearman correlation coefficients *r* are shown in the scatterplots. Data shown are from all three treatment groups combined. Small perturbations are added to titer values to improve visibility. Spearman correlation coefficients *r* are shown in the scatterplots. For the HAI titer plots, 8192 means >4096 and 4 means <8 (the upper and lower limits of quantification of the HAI assay were 4096 and 8, respectively)

As shown in Fig. 2, baseline HAI titers were higher (*p*<0.001) in the previously vaccinated whereas previous vaccination did not significantly associate with baseline NAI titers (*p*=0.16). In addition, baseline HAI titers were inversely correlated with age among previously vaccinated participants (*p*<0.001), but not among previously unvaccinated participants; moreover, baseline NAI titer was not correlated with age regardless of previous vaccination status (Fig. 2). We repeated these analyses for Day 30 HAI and Day 30 NAI, for each treatment arm (Additional file 1: Figures S4 and S5; Table S1). There were no significant differences in Day 30 HAI or Day 30 NAI titers by previous vaccination status within any arm. Day 30 HAI titer decreased significantly with age among the previously vaccinated in placebo recipients and among previously vaccinated in the IIV arm. Day 30 NAI titer decreased significantly with age among both previously vaccinated and unvaccinated placebo recipients.

### Baseline covariates as correlates of vaccine efficacy against influenza disease

Selected multivariable logistic regression models of VE against influenza disease that were functions of the baseline covariates Evervax (“previously vaccinated”), Day 0 HAI titers, Day 0 NAI titers, age, sex, and race are shown in Table 2 (one final model for each vaccine). Only variables with 2-sided *p*<0.05 in the multivariable model were kept in the final model, except vaccination status (Vacc) was kept. When an interaction term was added, the main effects that made up the interaction were also included in the model. These VE models were built upon separate correlates of risk models for individual treatment arms, in which we also tested for interactions between quadratic associations of Day 0 HAI and Day 0 NAI titers with influenza, as well as interactions between Evervax and Day 0 HAI titer and between Day 0 HAI and Day 0 NAI titers with influenza, and found no statistically significant evidence to add these terms to the models.

**Table 2:** Correlates of vaccine efficacy final logistic regression models based on baseline/pre-vaccination variables

	LAIV		IIV	
	OR (95% CI)	P	OR (95% CI)	P
Vacc	0.69 (0.38, 1.25)	0.224	0.05 (0.01, 0.28)	0.001
Evervax	0.70 (0.24, 2.05)	0.512	0.25 (0.03, 1.96)	0.189
Day 0 HAI	0.84 (0.74, 0.96)	0.007	0.71 (0.60, 0.84)	0.000
Day 0 NAI	0.60 (0.46, 0.78)	0.000	0.37 (0.20, 0.71)	0.002
Evervax:Day 0 NAI	1.69 (1.17, 2.43)	0.005	2.97 (1.30, 6.78)	0.010
Vacc:Evervax			16.81 (0.99, 284.65)	0.051
Vacc:Day 0 NAI			2.73 (1.31, 5.71)	0.008
Vacc:Evervax:Day 0 NAI			0.23 (0.08, 0.66)	0.006

^∗^All models adjust for age, sex and race at baseline. Vacc = 1 or 0 if assigned vaccine or placebo; Evervax = 1 or 0 if self-reported ever having an influenza vaccination yes or no; HAI and NAI titers are log base 2 transformed. ^∗∗^Entries for two-way interactions (e.g., Vacc:Evervax) are ratios of odds ratios, and for three-way interactions (Vacc:Evervax:Day 0 NAI) are ratios of ratios of odds ratios

**Fig. 3:**
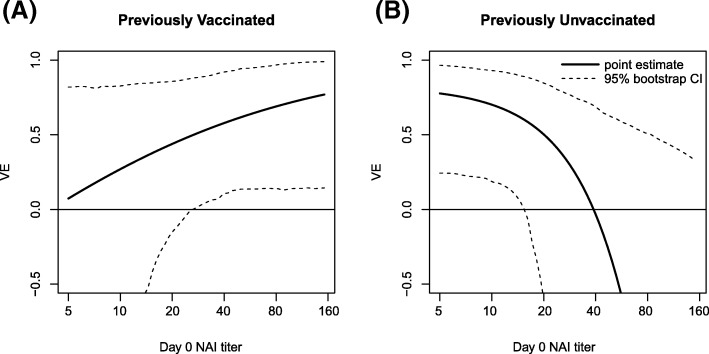
VE of the IIV vaccine depends on baseline NAI titer differently among the previously vaccinated and previously unvaccinated. VE-by-Day-0 NAI titer plots are shown for the IIV vaccine in (**a**) the previously vaccinated vs (**b**) unvaccinated

**Fig. 4:**
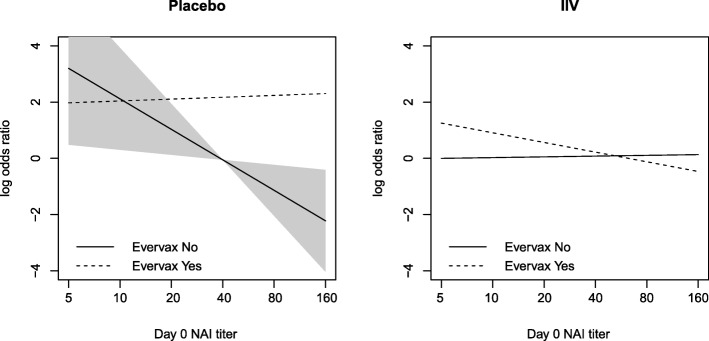
Evervax modifies the association between Day 0 NAI and influenza risk with decreasing strength in the placebo arm. The shaded area is the pointwise 95% confidence region for the risk function among Evervax=NO in the placebo group. The slopes of the other three risk functions are not significantly different from 0

Results from Table 2 show that VE of LAIV was not modified by any baseline variable. However, there was a significant interaction between IIV, Evervax, and Day 0 NAI (*p* =0.006); this interaction means that VE as a function of Day 0 NAI titer differs for the Evervax Yes and No subgroups. Figure 3 illustrates this three-way interaction by plotting estimated VE of IIV as a function of Day 0 NAI titer for the previously vaccinated and the previously unvaccinated, showing that higher Day 0 NAI was associated with increased VE in the previously vaccinated (estimated VE 7% at titer below the assay lower quantification limit and 57% at titer 40, *p* =0.23 for varying VE), but with lower VE in the previously unvaccinated (estimated VE 77% at titer below the assay lower quantification limit and 0% at titer 40, *p* =0.007 for varying VE). Figure 4 shows how this result seems to be driven by the previously vaccinated in the placebo group, for which influenza risk strongly decreases with Day 0 NAI titer. Results from Table 2 also show a highly significant inverse association between Day 0 HAI titers and influenza risk, without an interaction with Evervax or IIV. Additional file 1: Table S2 shows results of separate logistic regression model fits to each treatment arm.

### Day 30 and fold-rise HAI and NAI titers as correlates of vaccine efficacy against influenza disease

*Day 30 and Fold-Rise Correlates of IIV Efficacy.*

**Fig. 5:**
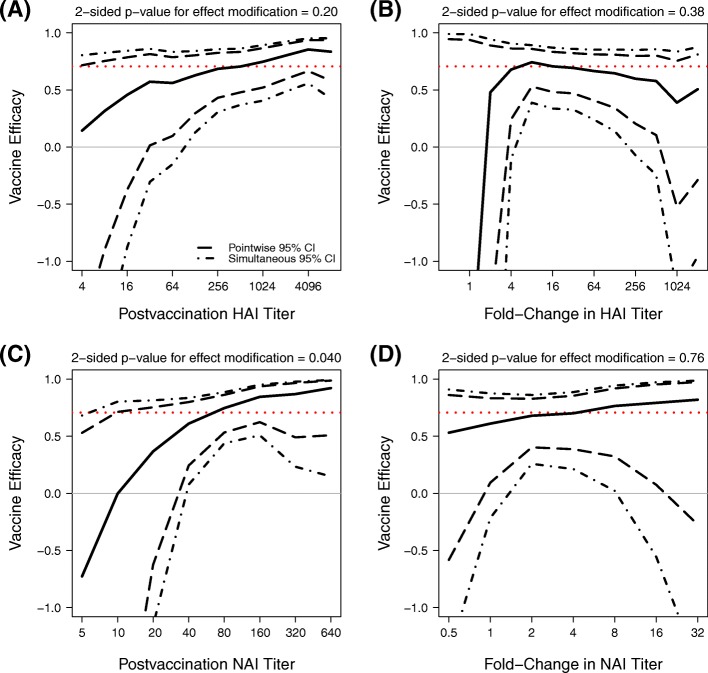
Estimated IIV VE by Day 30 and fold-rise HAI and NAI titer variables. Estimated VE by **a** Day 30 HAI titer if assigned vaccine, **b** fold-rise in HAI titer if assigned vaccine, **c** Day 30 NAI titer if assigned vaccine, **d** fold-rise in NAI titer if assigned vaccine. Each solid black line is the estimated VE by the Juraska (2018) method [48]. Dashed and dot-dashed black lines are pointwise and simultaneous 95% confidence intervals, respectively. Each dotted red line is the estimated overall VE. P-values shown are 2-sided p-values for effect modification

Estimated IIV efficacy as a function of Day 30 HAI titers in vaccinees and as a function of fold-rise in HAI titers in vaccinees, as well as the parallel results for NAI titers, are shown in Fig. 5. The estimated VE curves assess VE ranging over subgroups defined by a Day 30 biomarker measuring response to vaccination that is observable in vaccinees and a counterfactual that is predicted in placebo recipients; the method accounts for uncertainty in this prediction. Estimated VE increased with Day 30 HAI titer, albeit with nonsignificant evidence for titer-varying VE (*p*=0.20).

(Figure 5**a**). Moreover, estimated VE increased significantly with Day 30 NAI titers (*p*=0.040), with estimated VE above 80% at titers ≥ 160 and with estimated VE near zero at titer 10 but with low precision at lowest titers ≤ 20. Estimated VE was approximately constant with fold-rise in HAI titers and with fold-rise in NAI titers, with no evidence of titer-varying VE (*p*= 0.38, 0.76). We note there is strong evidence of high VE for vaccine recipients with high postvaccination HAI or NAI titers, based on the confidence bands that are well above 0, but there is large uncertainty about VE (as indicated by the wide confidence bands) for vaccine recipients with the lowest postvaccination HAI or NAI titers.

*Day 30 and Fold-Rise Correlates of LAIV Efficacy.*

**Fig. 6:**
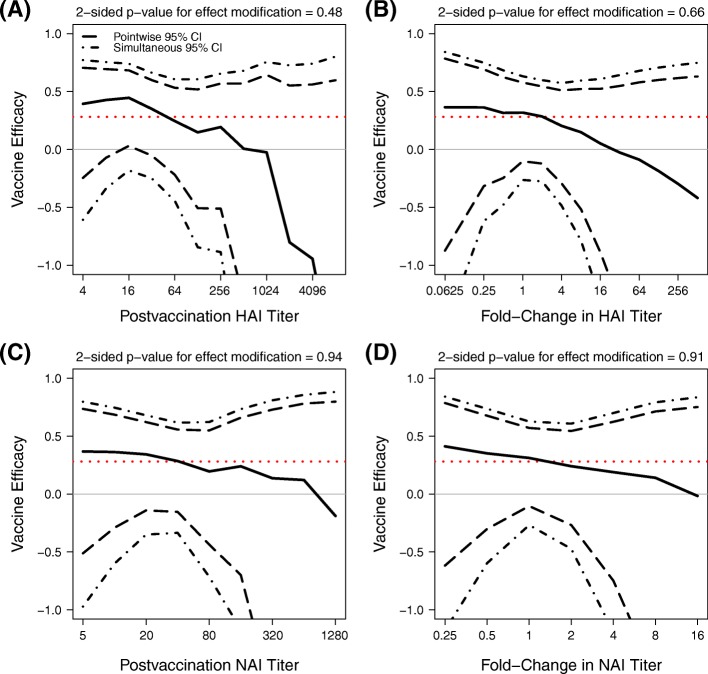
Estimated LAIV VE by Day 30 and fold-rise HAI and NAI titer variables. Estimated VE by **a** Day 30 HAI titer if assigned vaccine, **b** fold-rise in HAI titer if assigned vaccine, **c** Day 30 NAI titer if assigned vaccine, **d** fold-rise in NAI titer if assigned vaccine. Each solid black line is the estimated VE by the Juraska (2018) method [48]. Dashed and dot-dashed black lines are pointwise and simultaneous 95% confidence intervals, respectively. Each dotted red line is the estimated overall VE. P-values shown are 2-sided p-values for effect modification

For the LAIV (Fig. 6), there was no evidence for titer-varying VE by Day 30 HAI titer or by Day 30 NAI titer, (*p*=0.48, 0.94). There was also no evidence that fold-rise in HAI titer (*p*=0.66) or in NAI titer (*p*=0.91) associated with VE. The confidence bands generally include 0 for all marker subgroups and for each of the four markers, indicating limited precision in this study to learn about modifiers of LAIV VE.

## Discussion

Higher pre-vaccination/baseline HAI titers were observed among the previously vaccinated than among the previously unvaccinated, while no difference in NAI titers was observed. These findings suggest that NAI titers decay faster than HAI titers after vaccination or are not boosted as much by vaccination as HAI titers. The former explanation is unlikely considering that post-vaccination HAI and NAI titers have been observed to wane at similar rates [21, 51], while the latter explanation is supported by the fact that the NA concentration is not standardized in currently licensed influenza vaccines.

There was no evidence that LAIV efficacy depended on previous vaccination or baseline HAI or NAI titer. In contrast, IIV efficacy was significantly modified by previous vaccination and baseline NAI titers in an interactive way. In the previously vaccinated, estimated VE was near zero for individuals NAI seronegative at baseline and increased to about 75% for those with highest baseline NAI titers, whereas in the previously unvaccinated the opposite pattern was observed, with estimated VE about 75% for baseline NAI seronegative individuals and decreased to zero for those with baseline NAI titers at 40 or higher. This result seems to be driven by the placebo group, for which risk was not associated with baseline NAI titers in the previously vaccinated but was strongly inversely associated with baseline NAI titers in the previously unvaccinated. One possible explanation of this difference in risk profiles is that previously unvaccinated individuals with high baseline NAI titers represent individuals with sustained/durable NAI responses, perhaps obtained following natural infection, and there is lower risk of influenza disease. In contrast, for previously vaccinated individuals, the subgroup with high Day 0 NAI titers is a mixture of two subgroups: individuals who could have high NAI responses even without the previous vaccination, and individuals with a boosted NAI response from the previous vaccination, where the risk of influenza disease is not low. Alternatively, Day 0 NAI titers may lose their ability to accurately mark natural protection among individuals who have been previously vaccinated. The finding that previously unvaccinated individuals with high pre-vaccination HAI or NAI titers were unlikely to be protected by IIV may be related to multiple layers of protection conferred through naturally acquired immunity by prior influenza infection, operating not only through humoral responses but also possibly through cellular responses, which contrasts with the IIV-conferred protection that mainly operates via humoral responses to the HA and NA surface antigens. Moreover, the different result for the LAIV vaccine – no evidence of modification of LAIV vaccine efficacy by prior vaccination or by post-vaccination HAI or NAI titer – may strengthen this point, given that LAIV mimics natural immunity. Given the limited precision in the analysis, it would be important to confirm the effect modification findings in other studies. A caveat of this study was that data were not collected on recent influenza history, nor on recent vaccination, precluding the ability to study any moderating impact of recent natural or vaccine immunity on the HAI/NAI correlates of risk and of vaccine efficacy.

IIV efficacy significantly increased within strata of increasing Day 30 NAI titers observed for vaccinated individuals and predicted counterfactually for placebo recipients, and similarly seemed to increase with Day 30 HAI titers. In terms of point estimates, LAIV efficacy trended toward decreasing with both Day 30 NAI titers and Day 30 HAI titers, but with inadequate precision to infer a real decrease – overall there was no statistical evidence that LAIV efficacy correlated with any post-vaccination titer markers.

Mathematical modeling has been one approach to try to quantify the relationship between HAI titer and VE [3]. Such modeling has predicted that vaccines that elicit higher HAI responses should have higher VE against laboratory-confirmed influenza disease [52]. Influenza vaccine efficacy studies have also supported that post-vaccination HAI titers are positively associated with VE [12, 51, 53], although it has been proposed that such titers should not be used as a surrogate endpoint for reliably inferring VE due to the potential importance of cell-mediated immunity and anti-NA antibodies [12, 14]. Using the Prentice (1989) [54] surrogate endpoint framework with Dunning et al.’s (2015) scaled-logit model [55], Dunning et al. (2016) [56] analyzed a trial of the IIV vaccine at standard dose (as in FLUVACS) vs. high dose in persons 65 years of age and older and inferred that post-vaccination HAI titer of 40 corresponded to 50% protection (identical for vaccine- and natural-immunity for a valid Prentice surrogate) when the assay virus was antigenically well-matched to the circulating virus. Based on our VE moderation framework applied to FLUVACS, an HAI titer of 40 corresponded to estimated IIV VE of about 55% and an NAI titer of 40 corresponded to estimated VE of about 55-60% (Fig. 5), remarkably close to the previous estimates. Thus, use of the VE moderation framework may provide some additional confirmation that a post-vaccination titer response above 40 predicts reasonably high vaccine protection, complementing the result from the Prentice approach. As Dunning et al. did not analyze whether or how post-vaccination HAI fold-rise in titer corresponded to protection, the relative performance of post-vaccination absolute titer vs. fold-rise as correlates of protection in this framework cannot be compared.

The pattern of estimated VE as a function of Day 30 HAI titer in vaccinees was similar to that of the pattern with Day 30 NAI titer, and the analysis did not provide statistical support that one marker was a stronger correlate of VE. While our analysis lacked the statistical precision to discern which correlate was stronger, the fact that the point estimates showed a stronger association for NAI titer may suggest that NAI is as important as HAI. Had the association patterns been largely different by assay the study could have detected it– future studies with greater power would be needed to detect small-to-moderate differences. In addition, our inability to distinguish the predictive power of HAI and NAI titers may have been impeded to some degree by the modest correlation between these two antibody measures. Future studies could also assess how vaccine efficacy depends jointly on HAI and NAI titer levels, as there was insufficient sample size to support these analyses in the present study.

We next discuss some limitations of this work. First, as influenza vaccination was specifically recommended to individuals aged ≥50 years [57], ethical considerations precluded the enrollment of such individuals in the study, given that some of these individuals would have been randomized to placebo. Thus, we were unable to examine whether our findings held true in older individuals. This is an ongoing issue for all clinical trials of influenza vaccines, given the current recommendations. Given the evidence that T-cell responses may be a better correlate of protection against influenza disease than antibody titers in the elderly (≥65 years; reviewed in [58]), it is reasonable to hypothesize that we may see differences in the associations of HAI and/or NAI titer with VE, particularly in the elderly; however, there are considerable ethical and methodological challenges involved in testing this hypothesis.

In addition, a limitation of the VE moderation statistical methods used here is that they could give biased results if certain assumptions are violated [40, 46]. The methods assume that within each treatment arm the risk of the influenza endpoint conditional on the baseline and post-baseline biomarker follows a regression model, which can be directly checked. However, the methods also assume that, for placebo recipients, after conditioning on baseline demographic variables and the baseline and post-baseline biomarker values, influenza risk does not depend on the post-baseline biomarker value that would have been observed had the placebo recipient been vaccinated – this assumption cannot be validated because the vaccine response biomarker is counterfactual/missing. Undiagnosable violations of this assumption could cause bias in the estimated VE curves. A common misperception is that the VE moderation methods require perfect prediction of the counterfactual vaccine responses for placebo recipients; in fact, the methods were designed to account for uncertainty in the prediction and have been shown to provide valid results with partial predictiveness.

Another limitation of our analysis is that there were too-few influenza events to estimate VE by post-vaccination titer curves separately for the previously vaccinated and the previously unvaccinated. Given the significant interaction result that Day 0 NAI positively associated with IIV VE in the previously vaccinated yet negatively associated with IIV VE in the previously unvaccinated, and the positive association of Day 0 and Day 30 titers, we conjecture that the positive association of Day 30 titers with IIV VE may have been driven by the previously vaccinated subgroup. The design of future studies should consider planning for the enablement of such analyses with adequate statistical power.

## Conclusions

Our results suggest that future vaccine development efforts may consider prioritizing post-vaccination titer endpoints as criteria for screening and advancement of candidate vaccines, as better correlates of VE than fold-rise in titers. Moreover, they imply that for individuals with high HAI or NAI titers when the influenza season begins and who were not previously vaccinated, inactivated vaccination may be unlikely to confer protection beyond that already present due to natural immunity and/or immunity from previous vaccinations. Acknowledging the limited precision of the data set for studying moderators of vaccine efficacy, perhaps the most important conclusion is that in future vaccine efficacy trials it is critical to measure both baseline and post-vaccination titers, in both vaccinated and placebo recipients, to enable adequately precise estimation of the VE-by-postvaccination-response curves by leveraging the correlation of the Day 0 and Day 30 titers. Other baseline measurements could also help improve the precision in estimating the correlates of vaccine efficacy [59]. Few efficacy trials have measured the requisite data for estimating VE-by-postvaccination-response curves; an important conclusion is that future studies should plan such measurements.

## Additional file

Additional file 1 Tables S1-S2, Figures S1-S5. (PDF 322 kb)

## Abbreviations

HA: Hemagglutinin
HAI assay: Hemagglutination inhibition assay
IIV: Inactivated influenza vaccine
LAIV: Live attenuated influenza vaccine
NAI: Neuraminidase inhibition assay
VE: Vaccine efficacy
