# Supplementary material for: HAI and NAI titer correlates of inactivated and live attenuated influenza vaccine efficacy
Source: BMC Infect Dis. 2019 May 22;19:453. doi: 10.1186/s12879-019-4049-5 (PMC6530189; doi:10.1186/s12879-019-4049-5)
Supplement: Supplementary file 1 — Tables S1-S2, Figures S1-S5. (PDF 322 kb) [file 12879_2019_4049_MOESM1_ESM.pdf]

Supplementary Materials for "HAI and NAI titer correlates of inactivated and live attenuated vaccine efficacy in the FLUVACS trial"

Peter B. Gilbert[1,2*] Youyi Fong[1,2], Michal Juraska[1], Lindsay N. Carpp[1], Arnold S. Monto[3], Emily T. Martin[3], Joshua G. Petrie[3]

[1]Department of Biostatistics, Bioinformatics, and Epidemiology, Vaccine and Infectious Disease Division, Fred Hutchinson Cancer Research Center, Seattle, Washington, 98109, U.S.A.
[2]Department of Biostatistics, University of Washington, Seattle, Washington, 98195, U.S.A.
[3]Department of Epidemiology, University of Michigan School of Public Health, Ann Arbor, Michigan, 48109, U.S.A

*Corresponding author. Mail Stop M2-C200, 1100 Fairway Ave N., Seattle, Washington, 98109, U.S.A. Phone: (206) 667-7299; E-mail: pgilbert@scharp.org

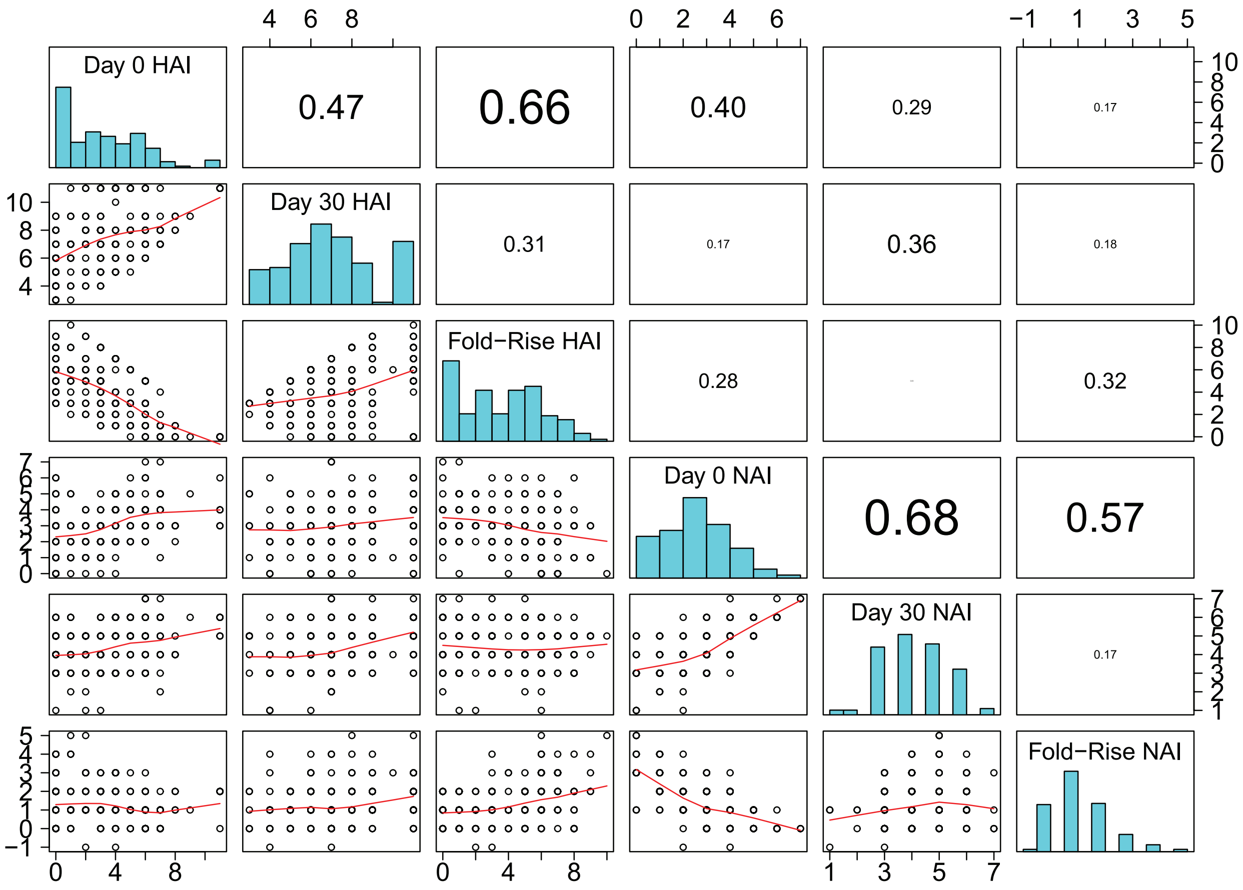

Figure S1: Distributions and scatterplots of $\log_2$ Day 0, $\log_2$ Day 30, and $\log_2$ fold-rise (Day 30 / Day 0) HAI and NAI titer variables in IIV vaccine recipients with complete titer data are shown. Numbers shown are Spearman rank correlations.

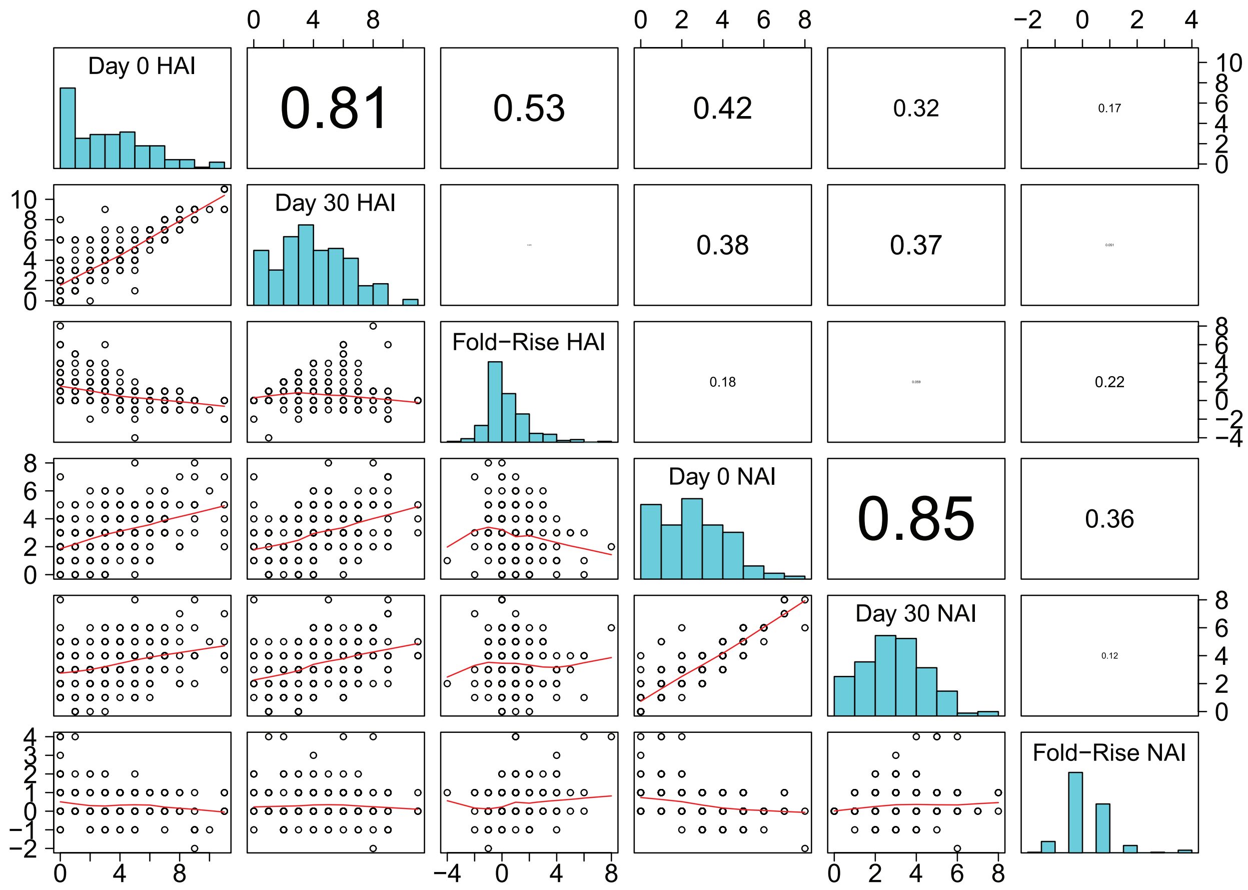

Figure S2: Distributions and scatterplots of $\log_2$ Day 0, $\log_2$ Day 30, and $\log_2$ fold-rise (Day 30 / Day 0) HAI and NAI titer variables in LAIV vaccine recipients with complete titer data are shown. Numbers shown are Spearman rank correlations.

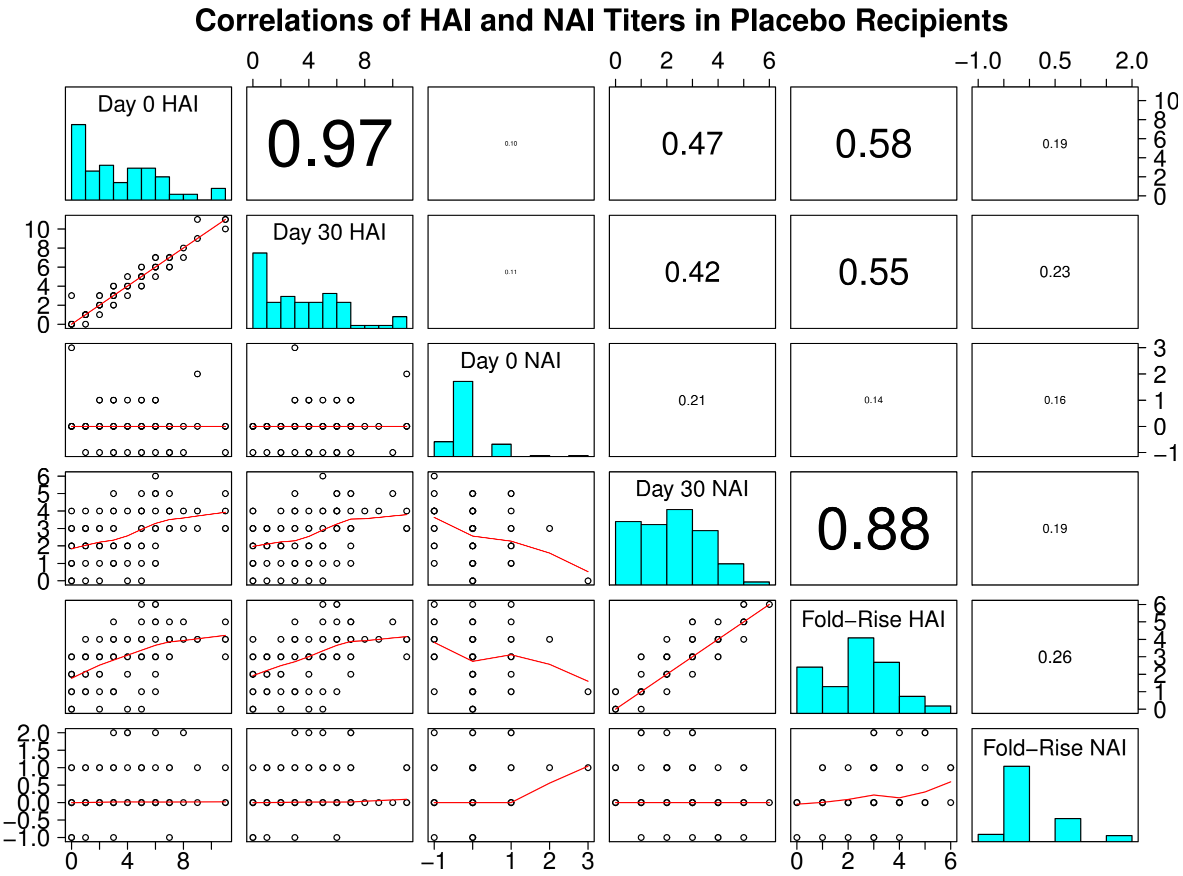

Figure S3: Distributions and scatterplots of Day 0, Day 30, and fold-rise (Day 30 / Day 0) HAI and NAI titer variables in placebo recipients with complete titer data. The numbers are Spearman rank correlations.

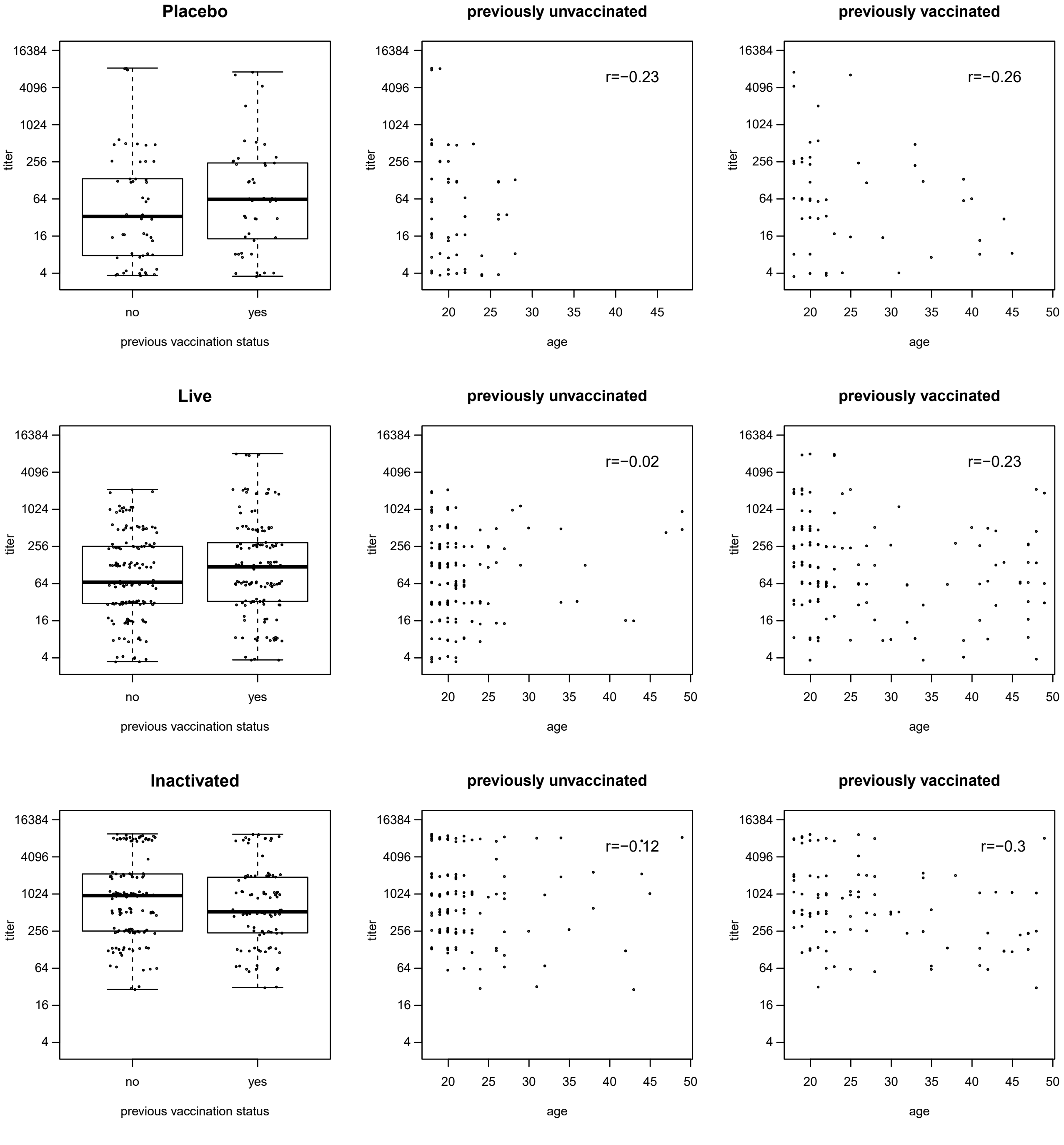

Figure S4: Boxplots of Day 30 HAI titers with and without prior vaccination.

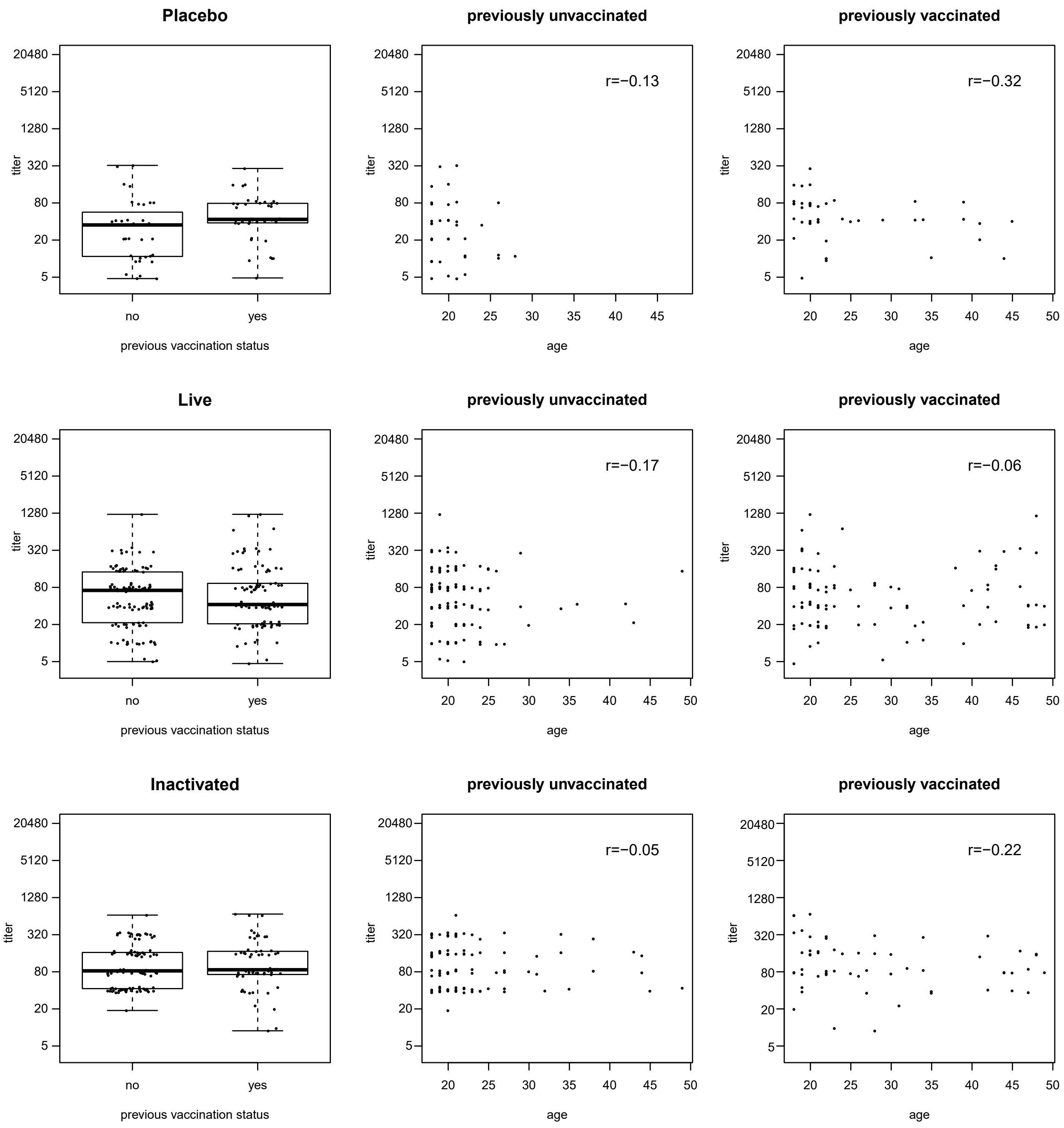

Figure S5: Boxplots of Day 30 NAI titers with and without prior vaccination.

Table 2 below shows correlates of influenza risk models for the IIV and LAIV groups that were built based on the same variables considered for the placebo arm in Table 1(A) and adding fold-rise NAI and fold-rise HAI (reported in Table 1(B)); the placebo arm is not considered for these models because fold-rise over 30 days does not measure a relevant biological response in unvaccinated individuals. For each vaccine arm we tested adding fold-rise HAI, the interaction between Day 0 and fold-rise HAI, the interaction between Evervax and fold-rise HAI, fold-rise NAI, the interaction between Day 0 and fold-rise NAI, the interaction between Evervax and fold-rise NAI, and the interaction between fold-rise HAI and fold-rise NAI.

For the LAIV, the fold-rise variables did not associate with influenza whereas Day 0 NAI and its interaction with Evervax had a similar pattern as in the placebo group, except the associations are weaker. For the IIV, all three variables Day 0 NAI, Day 0 HAI, and fold-rise in NAI were significantly inversely associated with influenza risk, and there were no interactions with Evervax.

We next repeated the correlates of risk models for the IIV and LAIV groups except adding the Day 30 NAI and Day 30 HAI instead of the fold-rise NAI and fold-rise HAI (reported in Table 1(C)). For the LAIV vaccine, the Day 30 variables did not associate with influenza, such that the selected model was the same as that in Table 1(B). For the IIV vaccine Day 0 HAI and Day 30 NAI were significantly inversely associated with influenza risk.

	titer∼evervax	titer∼age|unvaccinated	titer∼age|vaccinated
HAI			
Day 0	0.000	0.386	0.000
Day 30 Placebo	0.250	0.067	0.013
Day 30 Live	0.161	0.381	0.101
Day 30 Inactivated	0.121	0.661	0.005
NAI			
Day 0	0.159	0.193	0.443
Day 30 Placebo	0.224	0.042	0.022
Day 30 Live	0.901	0.322	0.958
Day 30 Inactivated	0.733	0.441	0.123

Table S1: P values associated with Figure 2 and Supplementary Figures 4 and 5. The tests are implemented using the R survey package, which uses model-robust variance estimates and accounts for two-phase sampling probabilities.

Table S2: Correlates of influenza risk logistic regression models based on baseline/pre-vaccination variables including previous vaccination status (Evervax), Day 0 HAI and NAI titers.

	Placebo		LAIV		IIV	
	OR (95% CI)	P	OR (95% CI)	P	OR (95% CI)	P
Evervax	9.25 (1.90, 45.09)	0.006	2.54 (1.07, 6.01)	0.035	1.15 (0.39, 3.43)	0.800
Day 0 HAI	0.69 (0.54, 0.88)	0.003	0.90 (0.78, 1.04)	0.171	0.73 (0.58, 0.92)	0.008
Day 0 NAI	0.34 (0.18, 0.64)	0.001	0.65 (0.49, 0.87)	0.003	1.03 (0.72, 1.47)	0.885
Evervax:Day 0 NAI	3.16 (1.30, 7.67)	0.011	1.51 (1.02, 2.25)	0.039	0.69 (0.37, 1.30)	0.254

*All models adjust for age, sex and race at baseline. Evervax = 1 or 0 if self-reported influenza vaccination for the previous flu season yes or no; HAI and NAI titers are log base 2 transformed.

**Entries for two-way interactions (e.g., Evervax: Day 0 NAI) are ratios of odds ratios.
